# Mad River Valley Dental Society

**Published:** 1884-11

**Authors:** 


### PROCEEDINGS.

### Mad River Valley Dental Society.

The society met in annual session in the parlor of the Phillips House, Dayton, Ohio, Tuesday, May 20th, 1884.

The President, A. BERRY, of Cincinnati, occupied the chair.

The subjects for discussion were as follows:

1. The Value of Pulpless Teeth and Roots.
2. Inflammation.
3. Alveolar Abscess.
4. The Promotion of Osseous Development.
5. Facial Neuralgia.

Papers were read by Dr. Geo. Watt, Xenia, on *The Promotion of Osseous Development*; *Antiseptics and Disinfectants*, by Dr. E. J. Lilly, Circleville, read by the Secretary; *Alveolar Abscess*, by Dr. F. W. Sage, Cincinnati; *A Plea for Education*, by Dr. Geo. L. Paine, Xenia; *A New Mouth Mirror*, by Dr. A. A. Blount, Geneva, Switzerland, read by Dr. Watt. Dr. Watt exhibited a porcelain gum tooth, made by Taft and Watt in 1852. It is the property of Dr. B. A. Satterthwaite, Dayton, who claims that the gum color is the most natural he ever saw.

A letter was read from E. H. Boune, D.D.S., Rocky Hill, N. J., requesting an honorary membership. The Secretary was instructed to notify the applicant that the society had no provision for any but active members, and they must be present at the meetings. It is peculiar in that it gets along without constitution, by-laws, or committees, and with very few officers.

The Secretary stated that the payment of dues for this year could be passed if the members in arrears would pay up.

W. A. Jackson (colored), of Dayton, was elected to membership. About twenty-five members were present at the meeting. Dr. Watt exhibited some vulcanized gutta-percha plates being probably the first ever used in the west. The case of E. A. Dill, of Yellow Springs, who is practicing illegally, was referred to the officers with power to act. The officers were instructed to prepare a programme for the next meeting. The papers read at the meeting were ordered published in THE DENTAL REGISTER, and the *O. S. Journal of Dental Science.*

Officers for the ensuing year: President, Geo. L. Paine, Xenia; Vice President, C. Bradley, Dayton; Secretary and Treasurer, W. H. Sillito, Xenia.

The thanks of the society were tendered Drs. Watt, Lilly, Sage, and Paine for their papers, also to Drs. Blount and Taft for their contributions.

THE VALUE OF PULPLESS TEETH AND ROOTS.

Dr. J. H. Paine felt better satisfied in retaining pulpless teeth in the mouth, than formerly. In many cases they did valuable service. The root having two sources of nourishment, the pulp and the periosteum, we can usually save it if the latter remains. The exposed pulp cannot live in an exposed condition. If the blood in it is a bright red we may be able to save the pulp by treating and capping; but if it has become dark and congested we destroy it with escharotics. Uses arsenious acid and glycerine; the former finely ground that it may be absorbed more readily. Now if we can fill the canal successfully and keep the periosteum healthy, the tooth will be useful for years. [In answer to questions by members.] Am not in favor of devitalizing pulps unless compelled to. Apply only a minute portion of arsenic. Many pulps destroyed with carbolic acid are only partly destroyed—the "legs," so to speak, are often left alive and the operation rendered uncertain. Strives to save pulpless teeth for people who will appreciate the service, even though they cannot pay full prices.

Dr. R. N. Boal—Why is it best to strive to remove all the pulp, if it is true that the "legs" may remain without giving trouble?

Dr. J. H. Paine—Because danger is always to be apprehended from a tooth with these amputated pulps.

Dr. J. Taft—I can remember, and it is not very far back, that if there was a pulpless root or an inflamed one in a mouth, the best method was thought to be to take it away, for fear it might do mischief at some future time; and often teeth were removed with the pulps alive. Have seen many cases where some crowns were gone; some decayed and some good, and they were all removed to make way for artificial dentures. Such would not be the treatment now. All teeth and roots that can be made useful, in any way, should be retained. They preserve the form of the features, and the expression for a great length of time. In a quarter of a century the practice has so changed that the removal of what was formerly regarded a nuisance would now be severely censured. The subject is receiving as much attention as any now before the profession. Some are practicing commendably in this respect, like the skillful surgeon who endeavors to save any part that can be restored to usefulness. I believe that the time will come when roots, such as are destroyed now by some of the most skillful, will be saved. If roots are allowed to remain they must, of course, be restored to health, or the teeth in their vicinity, being unused, will become foul and diseased. When roots are removed the change from loss of tissue extends to the exterior aspect of the face, and involves the voice also.

There are many methods of crowning, and none of them are good for all cases. Only by wide study and conscientious efforts will you succeed. If you fail do not give up, but try again. We cannot expect to succeed at once with a new method, although it may have great merit. Some take a liking to a particular method, persevere with it, and of course succeed. Sometimes a root will be rebellious, and we fail, but that should not discourage us. Physicians lose cases, so shall we be unsuccessful: we should go on in the expectation of success until we fail, and be exceedingly careful how we sacrifice teeth or roots that might be saved.

Dr. Geo. Watt—We always feel inclined to say "I told you so;" and some of you may remember how I was blackguarded (about 1857–8) for putting in a great many pivot teeth. A patient for whom I put in the six anterior superior teeth in 1858, lost them in 1876. My first case was two superior central incisors for a physician's wife. They were put in about 1853, and extracted in 1871. When the dentist came to extract for a full denture, he asked the patient how it was that the two front teeth were saved when the others were all decayed. They were the ordinary pivot tooth on a hickory pin. Dr. J. Taft filled a pulpless tooth for me 39 years ago, and although the crown is gone, the root still does service.

(A recess was taken to allow the examination of models and samples of crowns—the Howe, Bonwill, and others.)

Resuming the discussion:

Dr. J. Taft—If a pulp dies it must be without any assistance from me. A physician might as well decide to kill his patient because he thought he might not get well. Never use so deleterious an agent as arsenious acid.

### ALVEOLAR ABSCESS, FACIAL NEURALGIA, ETC.

Dr. J. H. Paine—First give ventilation by tapping the canal. Sometimes relieves periodontitis by raising the plate of the alveolar process, using a sharp instrument or blade. A case in practice is better than any theoretical case. A patient came to me, suffering from periodontitis. There was a large gold filling in the tooth. I did n't want to loose the tooth and filling. patient wanted it out. Extracted the tooth, removed the cyst, and replaced in its socket. The tooth is still good and serviceable after four years. I was careful not to remove the periosteum; most failures come from that. Out of eight cases of replantation, I have had six successful ones. Free bleeding by scarifying the gums and application of leeches always does good. Also paint the gums with equal parts tinct. aconite root and tinct. iodine. Hot applications and poultices should be avoided.

Dr. R. H. Boal—Do you fill the foramen?

Dr. J. H. Paine—No; just file off the spur; the rubbing closes it.

Dr. Boal—How many were lower teeth?

Dr. Paine—None; I recognize that it is harder to replant lower ones.

Dr. Boal—Have had aconite and iodine tried most effectually on myself. Never suffered so much in my life as from that application.

Dr. E. F. Sample—In cases of putrescent pulp, the gases penetrate the dentine, and before replantation the dead pulp, etc., should be removed, and the canal filled.

Dr. Paine—You misunderstood me; the pulp had been removed.

Dr. Sample—Apply on the skin over the sinuses a liniment composed: tinct. aconite root, $\frac{1}{2}$ ounce; chloroform and camphor, each, $\frac{1}{4}$ ounce. It gives immediate relief.

### THE PROMOTION OF OSSEOUS DEVELOPMENT.

Dr. J. H. Paine—In 1874 our only daughter, then a child, fell and broke her arm. We had then been using this kind of food mentioned in Dr. Watt's paper for several years. On the ninth day the family physician removed the splint to see if the apposition was correct. He found the bones already united, seemed astonished, and asked about the food. A mother asked if there was anything she could do to improve the teeth of the children. Recommended the brown flour pudding, etc. She used it, and at her next confinement the labor was easier, and the child's osseous development better.

Dr. E. F. Sample—We must admit that the teeth of the people are worse than the most avaricious dentist could desire. The past will not do for the present and future. We must do something beyond what our predecessors have done.

Dr. J. Taft—We too often discuss this subject as if all that was necessary was to have the deficient material. Look to the condition of the other tissues of the body, and put the system in good order. The soft tissues have a greater recuperative power than have the hard tissues; and there is less change going on in the teeth than in the rest of the osseous system, owing to their greater density, etc. Therefore the dentist should look further than the teeth, and study the growth and development of all parts of the body, and learn the physiological action necessary. It is not enough to give a bottle of lime phosphate occasionally.

I am in accord with the views presented in the paper of Dr. Watt. It is more important that we secure good teeth for the coming generations than to repair the decayed teeth of the present. It has been greatly neglected in the past. I think any mother would gladly take up and follow out the suggestions of an intelligent practitioner.

Dr. R. H. Boal—Am one of a family of three brothers and three sisters. I have the best teeth, and attribute it to my craving for Graham bread. The teeth of the other members of the family have had better care than mine have, and they have lost more teeth. Have had but seven filled.

Dr. A. J. Grosvenor—A few weeks ago, in examining the teeth of a patient, I found a filling undermined on one side with white decay. I told her the filling would have to be replaced, and she asked why my fillings were always dropping out.

She has lung trouble, and eats white bread and preserves, and does n't like oat meal, Graham bread, milk, or beef.

### MISCELLANEOUS.

Prof. Taft gave a most interesting lecture on inflammation, which we cannot give here.

Dr. A. Berry recommended asbestos felt as a superior packing for the vulcanizer.

The Secretary presented a pad of pure gum packing 3-16 in. thick, 5x4 inches, to be tacked over the edge of the bench, and used as a flexible cushion in filing plates.

Dr. J. Taft exhibited a superior third molar with a supernumerary in the bifurcation of the roots.

